# Supplementary material for: Suppression of Chitin-Triggered Immunity by a New Fungal Chitin-Binding Effector Resulting from Alternative Splicing of a Chitin Deacetylase Gene
Source: J Fungi (Basel). 2022 Sep 28;8(10):1022. doi: 10.3390/jof8101022 (PMC9605236; doi:10.3390/jof8101022)
Supplement: Supplementary file 1 [file jof-08-01022-s001.zip › jof-1924500-supplementary.pdf]

SUPPORTING INFORMATION

Table S1. Primers used in this study.

Figure S1. Nucleotide sequence alignment of *Podosphaera xanthii CDA* gene and derived cDNAs.

Figure S2. Predicted three-dimensional (3D) models of PxCHBE.

Table S1. Primers used in this study.

Primer name	Sequence (5´- 3´)[a,b,c]
***In vitro* expression**	
CHBEsexp-F	AAAAAGCAGGCTCTCAGAATACTTGCGGCCCAAGTATTGG
CHBE-STOP-R	AGAAAGCTGGGTGCTATTTGATGTCCACTGGTATAC
*att*B1	GGGGACAAGTTTGTACAAAAAAGCAGGCT
*att*B2	GGGGACCACTTTGTACAAGAAAGCTGGGT
**Translational fusions**	
PxCDA-F	ATGCAGATGTTAAATTTGGCACGTACGG
PxCDA1-noSTOP-R	GGTCCTCATCCGAAACCCTAAG
PxCHBE-noSTOP-R	TTTGATGTCCACTGGTATACATTCG
PgpdA-F	GAATTCTTAATTAAGATATCGAGCTCggtacc (KpnI)
RPgpdA-PxCDA1	**TACGTGCCAAATTTAACATCTGCAT**GGTGATGTCTGCTCAAGCGGGGTAG
Rgfp6-ApaI	gggcccTTACTTGTACAGCTCGTCCATGCCGTGAGTG (ApaI)
Fgfp6-PxCDA1	**ACCTTAGGGTTTCGGATGAGGACC**ATGGTGAGCAAGGGCGAGGAGCTG
Fcfp-CHBE	**CGAATGTATACCAGTGGACATCAAA**ATGGTTTCAAAAGGCGAAGAACTG
Rcfp-ApaI	gggcccTTACTTATAAAGTTCGTCCATGCCAAGTG (ApaI)
**Gene expression analysis**	
PxCDA/CHBE-F	ATGCAGATGTTAAATTTGGCACGTACGG
PxCDA1-R	TGTATAAGAGATGACCACTGAC
PxCDA2-R	TGTCCACTGGTATTTGTAGTGTC
CHBE-R	CTGGTATACATTCGTTTGTAGT
PxTUB2-F	GATTTAGAGCCCGGAACAATGGACG
PxTUB2-R	CACATCCTTCAGCTTCACGCCGAAC

[a]Underlined sequence is the adapter for *att*B1 or *att*B2 primers.
[b]Lowercase sequence denotes the recognition site for the restriction enzyme (indicated in brackets).
[c]Bold sequence denotes the tail of the other gene.

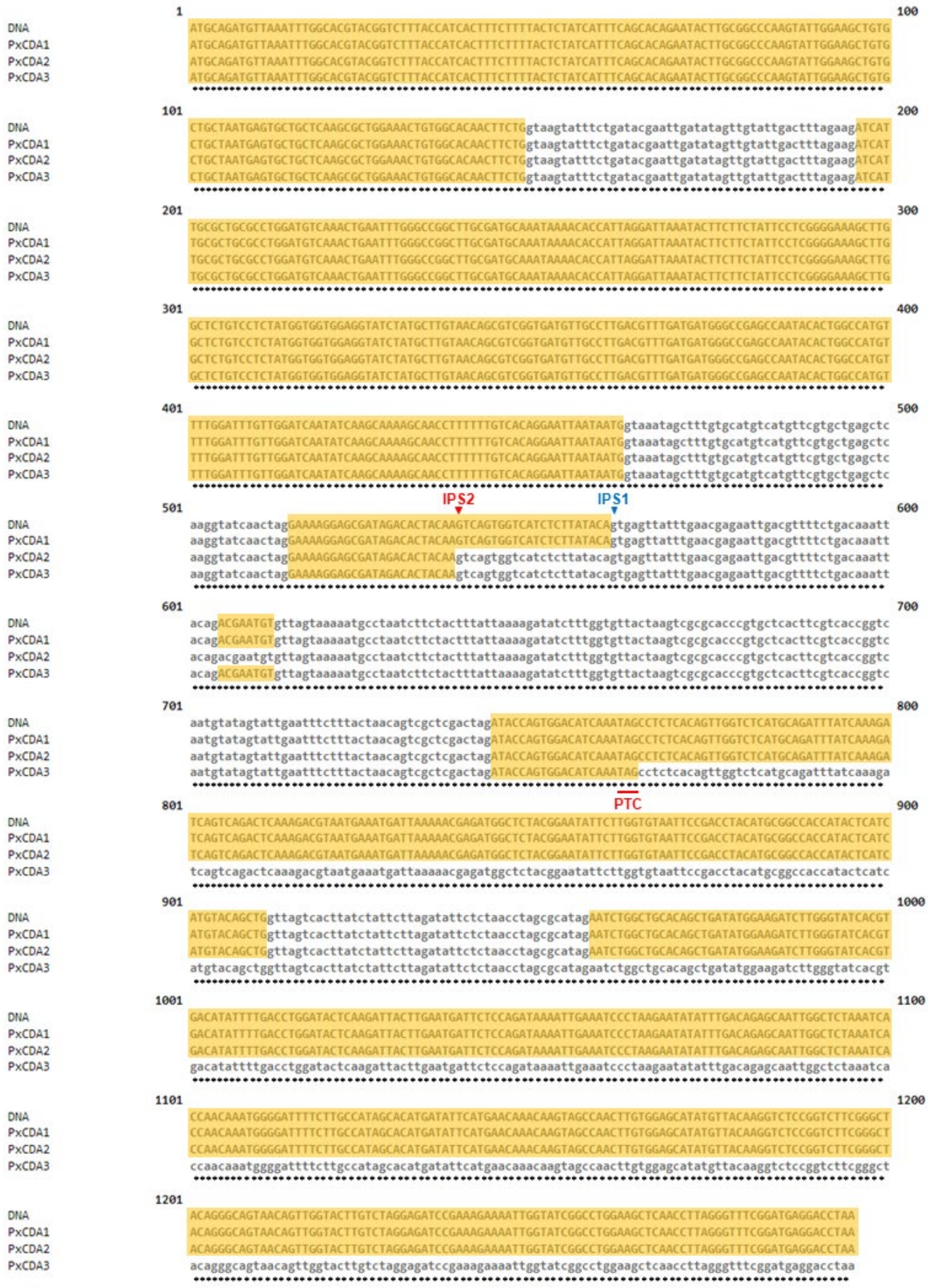

Figure S1. Nucleotide sequence alignment of *Podosphaera xanthii CDA* gene and derived cDNAs. The nucleotide sequences of the *PxCDA* gene (DNA) and the cDNAs of transcripts *PxCDA1*, *PxCDA2* and *PxCDA3* were aligned using MEGA 5 software. Translated sequences are shown in uppercase, while untranslated sequences are shown in lowercase. Abbreviations are: IPS, intron processing site; PTC, premature termination codon.

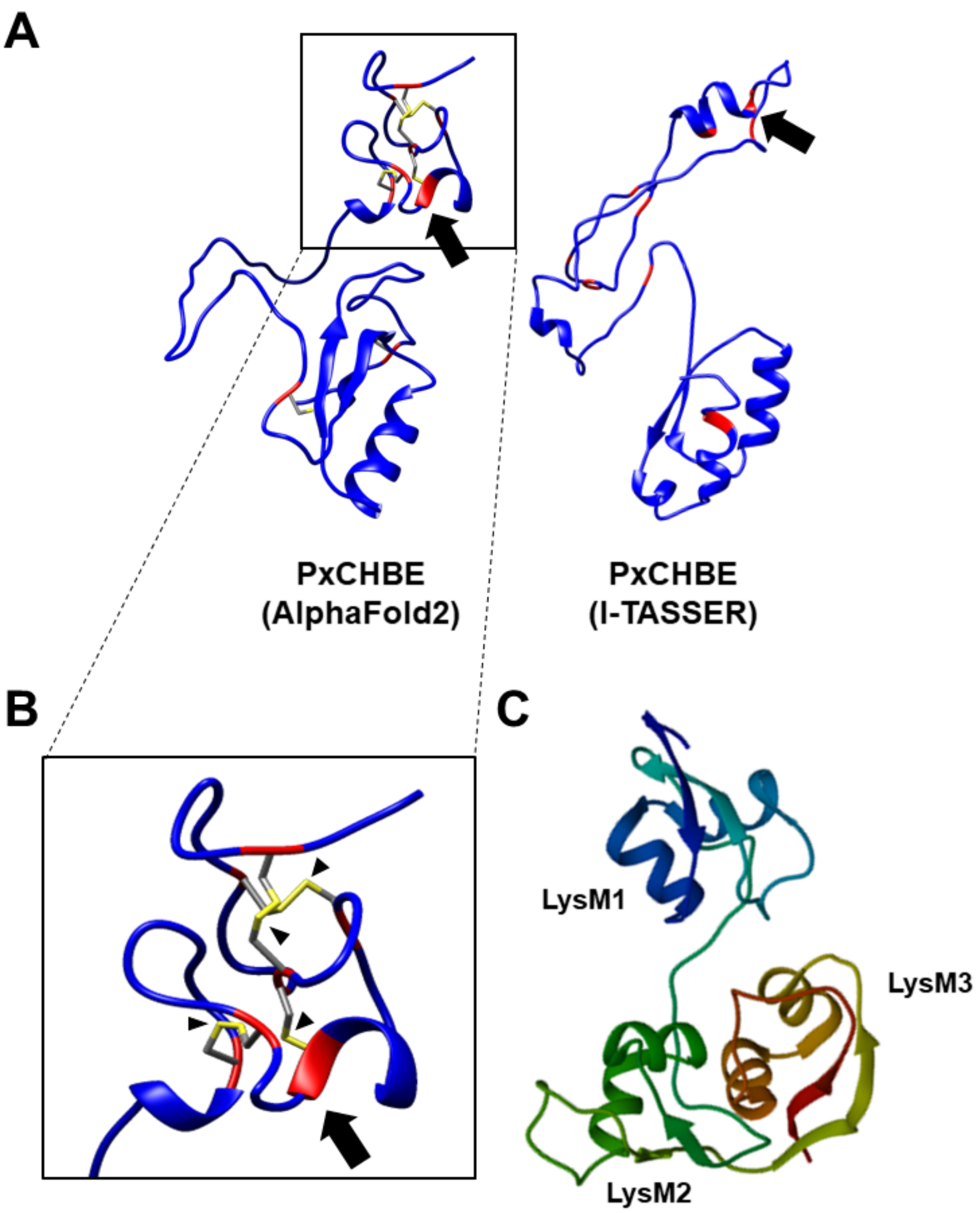

Figure S2. Predicted three-dimensional (3D) models of PxCHBE. (A) The 3D models were constructed using the AlphaFold2 and I-TASSER servers. The predicted chitin-binding domain of the PxCHEB protein models are indicated by arrows. Cysteine residues are depicted in red. (B) Detailed view of the region of the PxCHBE model enriched in cysteine residues predicted by AlphaFold2. Disulfide bonds are indicated by arrowheads. (C) The structure of the chitin-binding effector ECP6 from *C. fulvum* (CfECP6, 4B9H) obtained from the Protein Data Bank is shown for comparison. The three LysM domains of CfECP6 are also indicated. The LysM domain involved in chitin binding is composed of a pair of antiparallel beta strands separated by a pair of short alpha helices. Note how LysM domains are absent in the PxCHBE models.
